# Supplementary material for: Glucokinase activity suppresses hepatic cholesterol synthesis and triglyceride accumulation: A new model for the effects of the GKRP P466L common human variant
Source: bioRxiv. 2026 Jun 11:2026.04.07.717049. Originally published 2026 Apr 8. Preprint. [Version 2] doi: 10.64898/2026.04.07.717049 (PMC13081813; doi:10.64898/2026.04.07.717049)
Supplement: Supplementary file 1 [file media-1.pdf]

Reported Trait	p value	Beta	Odds Ratio	95% Confidence Interval	Citation
Triglycerides	1.5×10^{-9}				Orho-Melander et al. 2008 [19]
	5×10^{-4}		3.69	1.61 - 5.81	Vaxillaire et al. 2008 [11]
	2×10^{-239}	0.115			Willer et al. 2013 [11]
Metabolism-associated steatotic liver disease	1.06×10^{-10}		1.278	1.186 - 1.377	Anstee et al. 2020 [15]
Metabolism-associated steatohepatitis	3.78×10^{-7}		1.302	1.176 - 1.442	Anstee et al. 2020 [15]
Total Cholesterol	3×10^{-42}	0.051			Willer et al. 2013 [11]
HOMA-IR	5×10^{-5}				Orho-Melander et al. 2008 [19]
	4×10^{-6}		-5.45	-7.66 - -3.18	Vaxillaire et al. 2008 [11]
Fasting Insulin	5×10^{-5}		-3.9	-5.72 - -2.04	Vaxillaire et al. 2008 [11]
Fasting Glucose	9×10^{-9}		-1.27	-1.7 - -0.84	Vaxillaire et al. 2008 [11]

Figure S1: Summary of GWAS traits associated with rs1260326, the GKRP P446L variant

A.

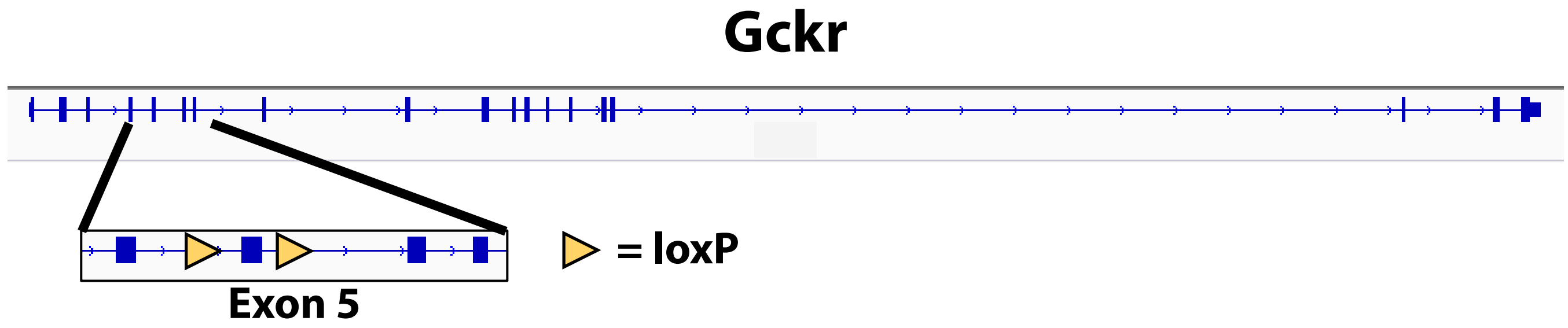

B.

ttctggaattttaatgaatagcttagtcagagaggatagtcaagaggatttctttctccagattactgttctttttgctacctcacct
ggcctatgctcgcctccattttatgatgatgagaagaagaaggtgttccaagtctccctctgacctgggcttctttccatcctca
ggagccagatgggggcctggtggtgctgagtggagggggcacctctggtcgtatggcattccttatgtctgtgagtacctta
attcctggcttctcccttctcctcctggggatagcccccagaacgaaaaggctcatctgaaataaggtctctcaatagatgtat
tctctccccacttcctctccaaaacctggagctcaggatggagtcagatttctctggccccactagagccccggcactctctt
gttaacatcctcacatcttgtgcatcactacgtaatcatttccaagttgcatctttaagtggcggactccaaacaagtataactt
cgtatagcatacattatacgaagttattagggttaagcattggcctaagaagaccattatgcaccttgatcctctaggacctg
agaacccgtgttatgtccttttttaatcactctgtcggcatattccctccccctggacagGTGTCTTTCAACCAGCTG
ATGAAAGGTCTGGGACAAAAACCTCTTTACACATACCTCATTGCAGGGGGTGACAG
gtaagttaccccgacctgtactcgtggcctgaatgttttttgtttgtttgtttgttttttgttttttttttaattgtatatgagtatactgtag
ctgtcttcagacataccagaagtgggcattggatcccattacagatgattgtgagccaccatgtggttgctggaaattgaact
cagtacctttggaggaacaatcagtgctcttaactgctgagccatctctccagcccccaaatgtctttttagaggctaaatttg
aatgaatttagtcaagcattatattgaacctgccttagtctccaacatcccataacttcgtatagcatacattatacgaagttatt
tgtggctttgtatctggccctgtcacagatgtttattgtgtgtcacgcctatcacagagcctgttaaacagtaggcgctgttcaat
gatagctttcctccatccctttctgttagtgtacctttcctaaccaccagtaggtatctgctccttacctaataccactgacctcgg
ctgagtttgcacccttaggtctgttgtagcctctcgggaacggacagaagatagcgccctacacggaatcgaggagctga
agaaggtctgtgtctcctgttactacttacctggagatgcccatatattcctttacatagggttagaaacccccaaccttcag
ccaaggcctggctcctgctctggtcctgattttctcacacactagatttgtaccctggagaccctaatgcacccctttcattccta
ggtggctgctgggaaaaagagagtggtcgttataggcatttccgtgggactctctgtaagtgtgaggtcaggaggggtg

Homology sequence

gRNA site #1: ggcggactccaaacaagtta

gRNA site #2: ttagtctccaacatcccttg

LoxP sequence: ataacttcgtatagcatacattatacgaagttat

Gckr Exon 5

Figure S2: Design of *Gckr* floxed mice:
A) Schematic of the mouse *Gckr* gene with exon 5 enlarged. Exon 5 was removed via CRISPR and replaced with a template containing loxP sites flanking it (detailed as triangles). **B)** Sequence for anti-sense template to create the floxed gene. The loxP sites were placed within the gRNA sites to prevent further Cas9 cutting once the template was incorporated into the genome.

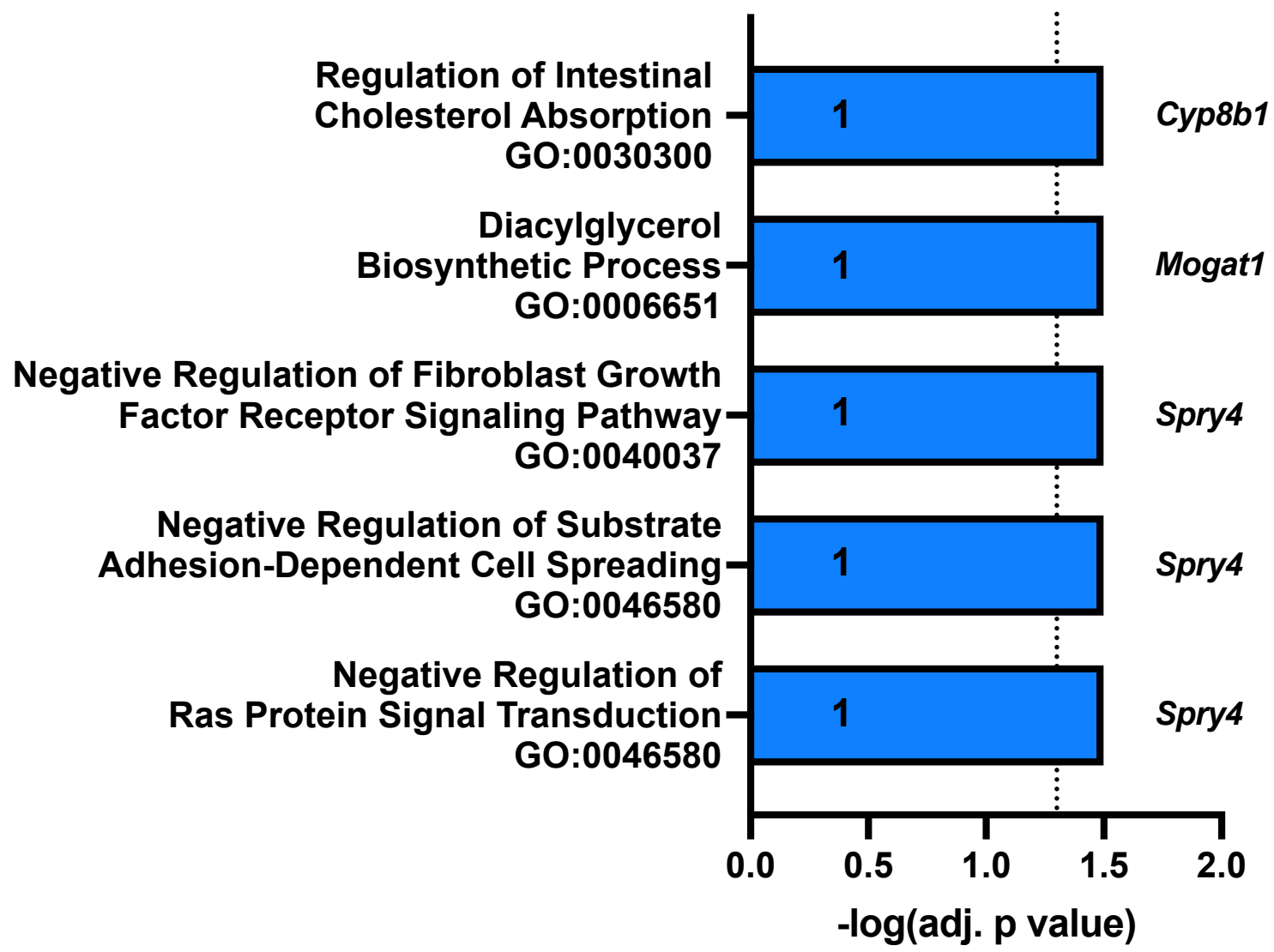

Figure S3: Top 5 downregulated gene ontology pathways from RNA-seq dataset (GO Biologic Process 2026). The number within each bar represents how many genes within each pathway were significantly down in the RNA-seq dataset. The list of those genes is presented next to each bar.
