# Supplementary material for: Glucokinase activity suppresses hepatic cholesterol synthesis and triglyceride accumulation: A new model for the effects of the GKRP P466L common human variant
Source: bioRxiv. 2026 Apr 8:2026.04.07.717049. Preprint. [Version 1] doi: 10.64898/2026.04.07.717049 (PMC13081813; doi:10.64898/2026.04.07.717049)
Supplement: Supplementary file 1 [file media-1.pdf]

Reported Trait	p value	Beta	Odds Ratio	95% Confidence Interval	Citation
Triglycerides	1.5×10^{-9}				Orho-Melander et al. 2008 [19]
	5×10^{-4}		3.69	1.61 - 5.81	Vaxillaire et al. 2008 [11]
	2×10^{-239}	0.115			Willer et al. 2013 [11]
Metabolism-associated steatotic liver disease	1.06×10^{-10}		1.278	1.186 - 1.377	Anstee et al. 2020 [15]
Metabolism-associated steatohepatitis	3.78×10^{-7}		1.302	1.176 - 1.442	Anstee et al. 2020 [15]
Total Cholesterol	3×10^{-42}	0.051			Willer et al. 2013 [11]
HOMA-IR	5×10^{-5}				Orho-Melander et al. 2008 [19]
	4×10^{-6}		-5.45	-7.66 - -3.18	Vaxillaire et al. 2008 [11]
Fasting Insulin	5×10^{-5}		-3.9	-5.72 - -2.04	Vaxillaire et al. 2008 [11]
Fasting Glucose	9×10^{-9}		-1.27	-1.7 - -0.84	Vaxillaire et al. 2008 [11]

Figure S1: Summary of GWAS traits associated with rs1260326, the GKRP P446L variant

A.

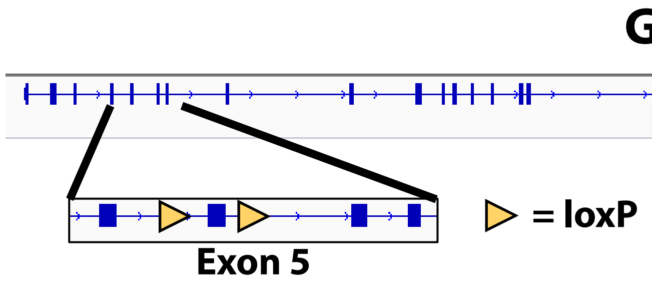

B.

ttctggaattttaatgaatagcttagtcagagaggatagtcaagaggatttctttctccagattactgttctttttgctacctcacct
ggcctatgctcgcctccattttatgatgatgagaagaagaaggtgttccaagtctccctctgacctgggcttctttccatcctca
ggagccagatgggggcctggtggtgctgagtggagggggcacctctggtcgtatggcattccttatgtctgtgagtacctta
attcctggcttctcccttctcctcctggggatagcccccagaacgaaaaggctcatctgaaataaggtctctcaatagatgtat
tctctccccacttcctctccaaaacctggagctcaggatggagtcagatttctctggccccactagagccccggcactctctt
gttaacatcctcacatcttgtgcatcactacgtaatcatttccaagttgcatctttaagtggcggactccaaacaagtataactt
cgtatagcatacattatacgaagttattagggttaagcattggcctaagaagaccattatgcaccttgatcctctaggacctg
agaacccgtgttatgtcctttttaatcactctgtcggcatattccctccccctggacagGTGTCTTTCAACCAGCTG
ATGAAAGGTCTGGGACAAAAACCTCTTTACACATACCTCATTGCAGGGGGTGACAG
gtaagttaccccgacctgtactcgtggcctgaatgttttttgtttgtttgtttgttttttgtttttttttaattgtatatgagtatactgtag
ctgtcttcagacataccagaagtgggcattggatcccattacagatgattgtgagccaccatgtggttgctggaaattgaact
cagtacctttggaggaacaatcagtgctcttaactgctgagccatctctccagcccccaaatgtctttttagaggctaaatttg
aatgaatttagtcaagcattatattgaacctgccttagtctccaacatcccataacttcgtatagcatacattatacgaagttatt
tgtggctttgtatctggccctgtcacagatgtttattgtgtgtcacgcctatcacagagcctgttaaacagtaggcgctgttcaat
gatagctttcctccatccctttctgttagtgtacctttcctaaccaccagtaggtatctgctccttacctaataccactgacctcgg
ctgagtttgcacccttaggtctgttgtagcctctcgggaacggacagaagatagcgccctacacggaatcgaggagctga
agaaggtctgtgtctcctgttactacttacctggagatgcccatatattcctttacatagggttagaaaacccccaaccttcag
ccaaggcctggctcctgctctggtcctgatttctcacacactagatttgtaccctggagaccctaatgcacccctttcattccta
ggtggctgctgggaaaaagagagtggtcgttataggcatttccgtgggactctctgtaagtgtgaggtcaggaggggtg

Homology sequence

gRNA site #1: ggcggactccaaacaagtta

gRNA site #2: ttagtctccaacatcccttg

LoxP sequence: ataacttcgtatagcatacattatacgaagttat

Gckr Exon 5

Figure S2: Design of *Gckr* floxed mice:
A) Schematic of the mouse *Gckr* gene with exon 5 enlarged. Exon 5 was removed via CRISPR and replaced with a template containing loxP sites flanking it (detailed as triangles). **B)** Sequence for anti-sense template to create the floxed gene. The loxP sites were placed within the gRNA sites to prevent further Cas9 cutting once the template was incorporated into the genome.
